# Preferences and Perceptions of Workplace Participation: A Cross-Cultural Study

**DOI:** 10.3389/fpsyg.2022.806481

**Published:** 2022-02-14

**Authors:** Sherry Jueyu Wu, Bruce Yuhan Mei, Jose Cervantez

**Affiliations:** Anderson School of Management, University of California, Los Angeles, Los Angeles, CA, United States

**Keywords:** employee participation, cross-culture, productivity, job satisfaction, conflict, norm, perception, China–US difference

## Abstract

Despite the amount of theorization on the forms and effects of participation, relatively little research directly examines what the concept of workplace participation entails in the minds of employees, and whether employees across cultures think positively when the concept of participation is activated in their mental representation. Three studies (*n* = 1,138 full-time employees) investigated the perceptions and preferences of full-time employees from the United States and China, cultures that might be expected to differ in their societal participation norm. Using a free association test and text analyses, Study 1 demonstrated that Chinese and American employees differed in their construal of workplace participation, yet both culture groups associated positive valence to the concept of participation. Study 2 showed that employees’ preference for workplace participation is positively related to their perceptions of its outcomes on productivity, job satisfaction, and workplace conflict. Study 3 had employees interact with either a prototypically high or low participation work environment and tested whether clear cultural contrasts might occur. American employees expressed unambiguous endorsement and predicted positive outcomes of a high participation workplace, whereas Chinese employees expressed slightly higher endorsement to a low participation work environment and associated it with higher productivity. This research provides insights on how workplace participation is construed by employees from different cultures, especially from cultures where democratic participation is not the normative default. Different perspectives on workplace participation across cultures may inform practitioners of the goals and approaches when shaping a more participatory workplace and a more democratic society.

## Introduction

Workplace participation is a common concept across multiple disciplines. For example, scholars in social and organizational psychology have focused on the role of worker voice and the design of teams in promoting individual workers’ productivity and morale (Lewin, 1947; Wu and Paluck, 2021). Scholars in industrial relations and labor studies have long studied different forms of representative participation, including co-determination and shared governance (Webb and Webb, 1902; Clegg, 1960). Political scientists have theorized the relationship between democratic practices that feature workplace participation and general civic and political engagement in the broader society (Pateman, 1970). Workplace participation is theorized at various levels of analysis by academics and practitioners (Wilkinson et al., 2010).

Despite the popularity of participatory strategies in the modern workplaces, relatively little is known about how the workers—or the targets of workplaces’ written or unwritten policies, processes, or regulations—come to understand and construe the concept of workplace participation (Greasley et al., 2005; Jeppesen et al., 2011). The present research shifts the perspectives from the academics and the management to the individual workers. We aim to address three primary research questions: first, how do workers construe workplace participation; second, how do workers perceive the effects of workplace participation on individual-level job outcomes such as productivity and job satisfaction; third, whether there is a cross-cultural preference for a participatory work environment. By addressing these research questions, we aim to shed light on the debate of whether employee participation reflects employees’ intrinsic motivation and provides positive outcomes from their perspectives, or whether it is simply an agenda that promotes the interest and values of the employers. We explore these questions through both surveys and experimental studies in the United States and China, two countries with drastically different cultures and political environments: namely, individualistic versus collectivistic, democratic versus nominally communist.

Below, we briefly review past research on workplace participation across different time periods and different disciplines as well as the proposed benefits and costs of participation, with a focus on research from psychology and behavioral science. We then move to the workplace contexts in the US and China, and develop our study hypotheses and experimental designs.

### The Employee Participation Debate: Forms and Meanings of Participation Across Disciplines and Times

Employee participation is an interdisciplinary topic that attracts interest from various social science disciplines, including psychology, economics, sociology, political science, among many others (Lucio and Stuart, 2005; Harley et al., 2010; Donaghey et al., 2011; Gollan and Xu, 2015). In psychology and behavioral science more broadly, researchers have defined “participation” as a behavioral process in which influence or decision power is shared between hierarchical superiors and their subordinates (Wagner and Gooding, 1987, p. 241). Defined psychologically, participation is a feeling of involvement in decision processes (Ritchie and Miles, 1970; Schuler, 1980; Miller and Monge, 1986). Extant research has used different meanings and forms of participation, as Heller et al. (1998) noted in their pioneering work at the Tavistock Institute:

In general the term refers to how employees are able to have a say over work activities and organizational decision-making issues within the organization in which they work. Some authors insist that participation must be a group process, involving groups of employees and their boss; other stress delegation, the process by which the *individual* employee is given greater freedom to make decisions on his or her own. Some restrict the term ‘participation’ to formal institutions, such as works councils; other definitions embrace ‘informal participation,’ the day-to-day relations between supervisors and subordinates in which subordinates are allowed substantial input into work decisions. Finally, there are those who stress participation as a *process* and those who are concerned with participation as a *result* (p. 15, emphases in original).

On the employee level, workplace participation takes various forms—it can be direct or indirect. A useful taxonomy was developed by Marchington and Wilkinson (2005), in which they differentiate employee participation into four forms: direct communication, upward problem-solving, representative participation, and financial participation. The first two forms (direct communication and upward problem-solving) are essentially direct and individual focused, oftentimes operating through direct interactions between supervisors and their staff. Some take the form of informal verbal communication, while others are through written information or suggestions. The third form (representative participation) focuses on the role of employee or trade union representatives in discussions between managers and the workforce via mechanisms such as joint consultation or joint working parties (e.g., Kessler and Purcell, 1996), and worker directors or collective bargaining (e.g., Perrett, 2007). The final form (financial participation) often operates through profit sharing or employee share ownership, whereby employees have a monetary stake or extrinsic benefit from their work beyond their salary. These various forms of participation differ in the scope of decisions employees are able to make, the amount of influence they can exercise over management, and the organizational level of analysis at which decisions are made. It is worth noting that the abovementioned taxonomy is not exhaustive. Beside those four forms, there are existing democratically structured enterprises that practice various forms of workplace participation on the organizational level, such as workers cooperatives and employee-owned firms (see Heller et al., 1998; Battilana, 2018; Weber et al., 2020).

It has been pointed out these forms of participation in focus vary across time and also interact with each other in a dynamic way (Deutsch, 2005; Wilkinson et al., 2010). New forms of participation emerge across different time periods, sometimes replacing their precedents while other times coexisting with prior forms of participation. The political and economic environments across different time periods have influenced the emergence and the spread of different forms of participation practices in the workplace, as well as the types of participation research that scholars work on in the academia (Wagner and Gooding, 1987; Deutsch, 2005).

The idea of workplace participation can be traced to as early as Rousseau and other political theorists on the role of industrial democracy and civic engagement (Rousseau, 1968; Pateman, 1970). Noticeably, there might be several societal changes that correlated with the development of different forms of participation research. First, the post-Second World War period witnessed a new era of worker participation, including the 1950s and 60s work at the Tavistock Institute in England, the Yugoslavian system of self-managing socialism, and systems of co-determination and representative workers’ councils and the rest (Deutsch, 2005). Parallel efforts from a research team led by Kurt Lewin, the founder of experimental social psychology, examined group decision making in the Harwood factory in West Virginia of the United States (Lewin, 1947). In the 1970s, the great amount of information flow and workplace innovations drove an enormous growth of participation research and practices in the Global North (Heller et al., 1998). After a major recession in the 1980s, issues of efficiency, productivity, and economic competitiveness—prominent challenges in a recession—became a primary focus in workplace participation. Changes in the nature of work—particularly from manufacturing to knowledge-based work and the shift toward non-unionism and individualism—saw workplace participation shifting focus again with “employee empowerment” becoming prominent in the 1990s and employee commitment and engagement taking over in the last decades (Wilkinson et al., 2010; Royle and Fox, 2011; Gollan and Xu, 2015).

Meta-analyses found a correlation between societal issues in the United States and the questions posed by American researchers, as well as a correlation between researchers’ social attitudes and their chosen methodologies of research (Wagner and Gooding, 1987; Crampton and Wagner, 1994). It was shown that the questions asked by American participation researchers mirror the trends of societal issues in the United States. From time-to-time different forms and meanings of participation became popular, even fashionable, and led to a proliferation of terms such as employee involvement, consultation, influence-sharing, decentralization, power-sharing, partnership, empowerment, and so on (Heller, 2003). While participation is the most commonly used term, its lack of clarity may lend itself to inconsistent applications and confusion over its definition. Thus, a potential weakness of the literature on employee participation is its lack of theoretical resemblance between studies that purport to measure or manipulate participation.

Furthermore, most of these participation related terms were coined by researchers interested in studying different aspects of participation. Much of the prior research focused on a deduction process—constructing or taking a form of participation in the workplace and studying its antecedents, outcomes, and evolution. Relatively little is known about how the concept of participation is construed in individual employees’ mental representation, and whether workplace participation, as understood by employees, is a good genuinely desired or is more a case of managerial agenda and intellectual debate imposed externally. Much of the literature assumes that employees desire participation and want a say in how they do their work, but some research suggests that we have overidealistic expectations and a tendency to implement participation measures through idiosyncratic social engineering efforts (Stein and Heller, 1979; Heller et al., 1998; Markey et al., 2013). While autonomy and social relatedness—important dimensions of participation and voice—is considered a universal need from a large amount of cross-cultural evidence (Deci et al., 2017), the desire for more influence or involvement might not be manifested in all contexts. Instead, employees’ desire for participation in specific issues may be determined by the congruence of the focal issue with their daily job functions, and whether a participation scheme is perceived to be genuine (Hespe and Wall, 1976; Liverpool, 1990; Kahnweiler and Thompson, 2000). Employees might not want more influence than what they thought they already had (e.g., voluntary non-participation, Heller, 1998); instead, employees’ social identification (Platow et al., 2015), specific organizational characteristics (Markey et al., 2013), and large societal cultures play important roles. For example, Jeppesen et al. (2011) investigated whether employees desire more participation and influence in their organizations by looking at who employees think should have more influence. They found that workers would want those already in charge of one area of work to have more influence in that area. In addition, their desire for influence was congruent with the influence they already have.

An interesting research question stemming from the literature on general workplace participation is given the current societal arrangements—the rapid pace of globalization, the trend toward knowledge-based work, non-unionism, and individualism—what forms of participation are salient from workers’ perspectives? We predict that individuals’ perceptions of workplace participation will vary based on societal prevalence of different participation forms. Given the trend toward non-unionism and individualism, direct participation—categorized as direct communication and upward problem-solving in Marchington and Wilkinson (2005)—should be better represented in employees’ mindset than less direct forms of participation such as representative participation and financial participation.

Relatively little research within psychology has directly examined what the concept of participation entails in the minds of employees, and what valence it elicits when the concept of participation is activated in individuals’ mental representation. Through qualitative research on four major construction projects, Greasley et al. (2005) illustrated a gap between management’s expectations and employees’ perceptions regarding workplace empowerment and pointed to the importance of exploring employee perspectives and the scarcity of this type of research. Khandakar et al. (2018) conducted 57 structured interviews in India’s banking industry and found a positive association between involvement in decision-making and the effectiveness of decision implementation and organizational performance.

The current research shifts perspectives from the academics or practitioners to individual employees who experience various forms of participation in their workplaces. Through free associations and text analyses, we elicit qualitative mental representations from employees and analyze the overarching content and valence associated with workplace participation using a mixed methods design. Recruiting full-time employees in different industries from the United States and China, we aim to provide insights on how employees define and understand workplace participation, and whether a cross-cultural preference for a participatory work environment exists.

### The Normative and Perceived Impact of Workplace Participation

The extant literature is predominantly Western centered, meaning the type of participation policies, processes, and experiences are primarily studied in the context of a Western democratic world (with the exception of Japan’s self-management practices pioneered in its automobile industry; it’s noteworthy Japan is still nominally a full democracy). Some argued that value-based endorsement of participation is a major contributor for participation research. Participation is a value rooted in Western political ideology. Modern notions of group participation echo 18th and 19th century western philosophical thought embodied in writings from Rousseau and John Stuart Mill, who argued that participation serves an educational function and trains individuals to be responsible citizens (Pateman, 1970). However, participation may not be universally valued in the structure of societies’ workplaces and institutions. For instance, in some East Asian cultures, endorsement of strict social hierarchies featuring minimal participation is prevalent in philosophical literatures. As a particular case in point, Confucianism denotes a basic set of moral principles for women (Three Obediences and Four Virtues), which emphasize absolute obedience for females to their male counterparts. Even though these behavioral codes have been abandoned, they have shaped East Asian cultures to the extent that harmony and obedience might be prioritized over participation and dissent (Landry, 2008; Truex and Tavana, 2019). Thus, an investigation on the perceived benefits of participation is necessary in non-Western societies. Do cultural beliefs in the benefits of participation vary? Or is participation perceived as a more universal good, and just used to differing degrees across different human contexts?

The perceived benefits, or outcomes of workplace participation, are featured prominently in debates (Kessler and Purcell, 1996; Harley et al., 2010; Knudsen et al., 2011; Gollan and Xu, 2015). Participation researchers and practitioners have used various outcomes to assess the impact of participation. From an organizational perspective, research has focused on short-term impacts on productivity or profitability, while some have adopted longer-term measures such as the well-being of and the trust between management and labor (Kessler and Purcell, 1996). Recent field experiments also found that participation in local work groups has spillover effect on employees’ outlook toward the society including reduced authoritarianism and belief in a just world (Wu and Paluck, 2020).

In psychology and behavioral science, a number of theories exist to explain the benefits of workplace participation. First, participation may flatten social hierarchy in one’s local group and the organization, by sharing influence, decision power, or more general involvement between group members and supervisors. A less hierarchical group structure may reduce conflict among different group members and therefore increase performance (Bunderson and Reagans, 2011; Greer et al., 2017). Second, participation amplifies information sharing and builds competence for individual employees (Locke and Latham, 2002; Heller, 2003). Lastly, participation increases opportunities to voice one’s perspective in decision-making processes (Vroom and Yetton, 1973). The direct experience of having one’s voice heard by group members may be a motivational force for behavioral change (Tyler and Blader, 2003; Wu and Paluck, 2021).

Relatedly, several prominent psychological theories such as the self-determination theory and procedural justice theory suggest a positive perception of workplace participation and its outcomes. Self-determination theory (SDT) postulates that individuals have innate psychological needs for competence, autonomy, and relatedness (Deci and Ryan, 2000; Deci et al., 2017). These needs are likely to be influenced by participatory decision making at work because participation in group decision-making allows employees to feel that work goals and outcomes are within their control as they actively involve themselves in the decision-making process, compared to when a decision is handed over to them. This is consistent with procedural justice theory as well—when individuals participate in a decision-making process, they can observe the contingency between goal setting and its completion, increasing the perceived procedural justice of the decision process (Thibaut and Walker, 1975; Tyler and Blader, 2003). Consequently, employees may also increase their appreciation for the workplace authorities that facilitate the participatory experience and increase their decision satisfaction (Tyler and Lind, 1992). Indeed, there is consistent support for the positive relationship between participatory decision-making at work and job satisfaction (Miller and Monge, 1986; Foels et al., 2000).

Meanwhile, a cluster of factors explain the cost of participatory practices (Jensen and Meckling, 1976; Hanushek et al., 2013). One commonly cited explanation is the misalignment of incentives between principals (e.g., employers) and agents (e.g., employees) in organizations (Jensen and Meckling, 1976; Dessein, 2002; Bandiera et al., 2021). Allocation of authority to the agents might exacerbate opportunistic behavior, responsibility shirking, and agency conflict (Jensen and Meckling, 1976; Mansuri and Rao, 2013). Furthermore, it might enable workers to coordinate and protest for demands such as a fair wage and better working conditions, which employers might not value to the equal extent. Some research demonstrates the tendency of marginalizing employee participation due to employers’ dominant concerns of its negative impact on efficiency or on economic growth (Kessler and Purcell, 1996).

Empirically, the benefits and costs of workplace participation vary depending on the specific outcomes measured. A study surveyed American full-time employees on their predictions regarding participation, productivity, and job satisfaction (Wu and Paluck, 2021). Over half (55.3%) indicated that a high-participation group structure in which “workers discuss work strategies in an open discussion and set goals for themselves” would be *less productive* compared with a low-participation group structure in which “a supervisor talks about work strategies in a lecture and set goals for each worker,” while the majority did predict that the high participation group would be more satisfied at work. The effects of participation, broadly defined, on group members’ behavior vary from positive to null and even some negative effects (e.g., Latham and Yukl, 1976; Richter and Tjosvold, 1980; Schuler, 1980). According to meta-analyses, some of the inconsistent findings for the effects of participation on behavior can be attributed to methodological variations. Strong correlations between participation and behavior seem to rely on individuals’ self-reports: *r* = 0.39, while studies that measure participation or behavior with multiple methods reveal a small average correlation of *r* = 0.12 (Wagner and Gooding, 1987; Crampton and Wagner, 1994). However, using objectively measure productivity and participation behavior, a recent large-scale field experiment (Wu and Paluck, 2021) demonstrates that a participatory vs. a hierarchical group meeting structure caused a 10.6% increase in worker productivity, which endured for an extended period of time even after the experiment ended. Participatory meetings also increased treatment workers’ retention and feelings of empowerment such as job satisfaction and sense of control. Experimental evidence in the field is valuable in estimating the causal direction as well as the effect magnitude of participation schemes in different cultural contexts.

Another contested question pays attention to the legitimacy of workplace participation: whether workplace participation really provides employees increased voice and well-being, or whether it is simply a managerial agenda that primarily promotes the interests of employers. In other words, instead of a “contested terrain,” is workplace participation more of a “captured terrain” (Gollan and Xu, 2015, p. 9)? We try to investigate this question by shifting perspectives from a top-down deduction to a bottom-up induction process, starting from the workers’ point of view. Certainly, there are limitations to this approach that we will discuss at the end of the paper, but this will give us a fresh perspective on the debate. That is, from a worker’s point of view, do they value workplace participation as a driver of productivity and well-being, or more of a managerial agenda that deviates from the workers’ interests? We are particularly interested in employees’ expectations of the outcomes from participation: whether employees across cultures regard workplace participation as a boon or a bane for their performance and well-being, especially from cultures where democratic participation is not the normative default.

Specifically, we will investigate employees’ perceived outcomes of workplace participation, focusing on three important dimensions: productivity, job satisfactions, and conflict. Productivity refers to the efficacy of workplace participation stemming from the perspectives of both the management and employees. Job satisfaction represents employees’ affective response, which serves as an umbrella construct covering the perceived quality of the work environment and culture, and employee interest and well-being. The first two measures are commonly studied in the past literature as primary outcomes of workplace participation. Past research argues that satisfaction levels might be a more proximal indicator of workplace participation, while productivity might be a more distant indicator, which depends on the interplay of other individual and group level factors, such as job demands and resources (Demerouti et al., 2001). In the present research, we include a third dimension—workplace conflict, or the antithesis of harmony. On one hand, research suggests that a flatter hierarchy and higher level of group participation might facilitate organizational performance through reduced intragroup conflict (De Wit et al., 2012; Greer et al., 2018). On the other hand, some have suggested the conflict between authentic participation and efficiency: managers may embrace the idea of participation because it has become a “popular mythology” (Heller, 2003, p. 147), but acting on it may engender their managerial prerogative and induce workplace conflict (Argyris, 1993). We adapt our survey measures from established scales on perceived productivity, job satisfaction, and workplace conflict—three potential indicators of workplace participation.

We explore employees’ preferences and perceived outcomes of workplace participation by focusing on two contrasting cultures—the United States and China. The United States and China are known to be distinct on various dimensions: West and East, individualistic and collectivistic, independent and interdependent cultural orientations, democratic and nominally communist (Nisbett and Masuda, 2003; Wu et al., 2018). As major competitors in today’s global world, the United States and China have differing ideologies, in the past and in the present, even if both now function as market economies. The two societies seemingly differ, and little empirical participation research has ventured out of the Western democracies—but how do they vary on employees’ preferences and perceptions of workplace participation?

### Review of Hypotheses

The current research aims to investigate the preference and perceived outcomes of workplace participation among employees in mainland China and the United States.

We made the following hypotheses based on theories and empirical evidence:

Hypothesis 1: Employees differ in their mental representations of what workplace participation generally means. Because China and the United States differ in the prevalence of democratic and workplace participation of their citizens and employees, we predict that individuals’ mental representations of workplace participation may reflect the societal norms and thus will differ across cultures.
Hypothesis 2: In both cultures, preference for workplace participation predicts perceptions of (a) increased productivity, (b) job satisfaction, and (c) reduced workplace conflict. According to Weber et al., 2020’s meta-analysis, employees’ perceived participation in organizational decision-making is positively related to a series of psychological outcomes such as job satisfaction, work motivation, prosocial work behaviors, among many others. Therefore, we predict employees’ preference for workplace participation is related to similar psychological and performance outcomes.
Hypothesis 3: Chinese employees show a stronger belief that workplace participation will undermine worker productivity and increase conflict than American employees. Hypothesis 3 is exploratory as there is little systematic cross-cultural research on the *perceived* effect of workplace participation on conflict. From prior qualitative work of the first author of the paper, it was not uncommon for supervisors in China to describe outspoken workers as “trouble-makers” who would introduce conflict and disrupt a “harmonic” environment of a work team. While such perception might not reflect the reality on how assertive workers influence group dynamics, the perception of participation as an obstacle to group cohesion could negatively impact the adoption of workplace participatory schemes and therefore is worth exploring. Here, we make this preliminary prediction, acknowledging its purely exploratory nature.
Hypothesis 4: Employees in both cultures generally prefer a participatory work environment to a non-participatory work environment. The desire for more participation and influence at work may not be always applied to every single domain (Jeppesen et al., 2011). However, because a participatory work environment is likely to increase one’s sense of relatedness and autonomy, which is deemed as fundamental needs across cultures (Deci and Ryan, 2000; Deci et al., 2017), we predict that employees *in general* prefer participation to non-participation as workplace norms.

### Current Studies

Three studies test these hypotheses. Study 1 administered a word-association test and asked employees in mainland China and the United States to spontaneously generate as many concepts (e.g., words, phrases, and emotions) as possible associated with workplace participation. We conducted text analyses using Linguistic Inquiry and Word Count (LIWC) as well as manual coding by three independent coders to investigate the content and valence associated with workplace participation from an employee’s perspective. Text analysis provides an efficient method for studying the various emotional, cognitive, and structural components present in written samples (Tausczik and Pennebaker, 2010). Content word categories explicitly reveal where individuals are focusing, which would provide key insights for Hypothesis 1. Study 2 assesses employees’ preference for workplace participation and its outcomes with Likert-scale items, testing hypotheses 2 and 3. We have adapted and cross-validated these survey items in both cultures to measure preference and three dimensions of participation outcomes: productivity, job satisfaction, and workplace conflict. Study 3 is a within-subject experiment that randomly assigned participants into a hypothetical participatory work environment and a non-participatory work environment in order to elicit their perceived desirability of working in each work environment and the downstream attitudes of participation at work, testing hypothesis 4. Studies 2 (China data collection) and 3 were pre-registered.

This research should add to our understanding of employees’ spontaneous preferences and associations of workplace participation, and shed light on the use of participatory work practices as a change vehicle to both workplace behavior and attitudes. First, negative perceptions of workplace participation effects, if any, may reveal obstacles of using workplace participation as an intervention to improve company and individual performance, especially in non-Western cultures. Second, different perspectives on workplace participation across cultures may also inform practitioners of the goals and approaches they may prioritize when shaping a more participatory work environment and a more participatory society with an increasingly diverse population.

## Study 1

Study 1 investigates the spontaneous associations employees make when they think about the concept of workplace participation and uncovers any valence behind the automatic associations.

### Method

#### Participants

Study 1 was a descriptive study. We aimed for 150 full-time employees from each culture; a total of 360 full-time employees across different industries in both the United States and mainland China participated in the study. American employees (*n* = 149) were recruited from Amazon Mechanical Turk on May 18th, 2021 (40.27% women and 59.06% men; *M*_*age*_ = 38.56 years, *SD*_*age*_ = 11.97, *range* = 18–80 years; 73.82% identified as White or European American, 8.72% Black or African American, 7.38% as Asian or Pacific Islander, and 4.03% Hispanic). We only recruited participants who were currently employed in a full-time job (over 35 h per week). Chinese full-time employees (*n* = 211) were recruited on June 7th, 2021 from Wen Juan Xing, an online crowdsourcing platform in mainland China that provides functions equivalent to Amazon Mechanical Turk (55.92% women and 44.08% men; *M*_*age*_ = 32.94, *SD*_*age*_ = 7.59, *range* = 21–65 years). Employees were compensated for their participation in the study (see Supplementary Appendix Table 1 for detailed demographic summary for the cross-cultural sample for all three studies).

#### Procedure

Employees completed a free-association task where they made free associations with the concept of workplace participation. Specifically, they were asked to write down as many words, phrases, or sentences that immediately came to their mind while thinking about worker participation (*员工参与* in Chinese), based on their first thoughts, beliefs, experiences, or impressions related to the concept. After the free association task, we collected standard demographics information including gender, age, race and ethnicity (in the United States), education, occupation, and general political ideology.

All participants were tested in their native language. The study material was first developed in English and then translated (with back translation) into simplified Chinese. Two bilingual researchers cross-checked to make sure the survey content was equivalent across languages. This procedure was followed for all studies reported in this paper.

### Results

#### Overarching Categories

By a word count, American participants (*M* = 17.73, *SD* = 13.51^1^) wrote longer than Chinese participants did (*M* = 12.70, *SD* = 11.74), which could be a proxy for the number of associations generated. To better understand the semantic content associated with workplace participation, three independent research assistants (including two bilingual English–Chinese speakers) clustered all the associations into major overarching categories by grouping words and phrases with similar meanings together (see Table 1; refer to Supplemantary Appendix B for details on the coding procedure). Then two bilingual coders coded each participant’s responses into these categories. The percentage of agreement between the coders was 94.0% (α = 0.78) for the Chinese data and 94.4% (α = 0.74) for the English data (Krippendorff, 1980).

**TABLE 1:** Major categories of associations with workplace participation in American and Chinese samples.

Major category	Scope	Example responses
Extrinsic	External rewards or costs of participation	Money, time cost, what it takes, and how much paid. 发奖励, 提高工资, 持股, 分红
Teamwork	Teamwork, group dynamics, concepts of sociality or collectiveness	Working together, teamwork, and cooperate. 合作, 集体活动, 团队
Negative valence	Words of negativity or disproval	Annoyed, lazy, stressed, overworked, and low-paid. 有内幕, 被动, 很反感, 忽悠
Ideology and hierarchy	Workplace hierarchies, high-level work ideologies; politics-related concepts (in China)	Equality, democracy, hierarchy, and moving up the ladder. 民主, 平等, 员工和管理层, 投票选举
Positivity	Universally positive traits; positive and encouraging language	Active, responsible, friendly, and integrity. 开心, 主动积极, 进取, 有收获
Socializing outside of work (unique to China)	Behaviors or activities shared by the work team outside of the work context	(Casual chatting, team development, traveling, picnic, and games). 社交聊天群, 团建, 旅游年会, 聚餐玩游戏
Management rules (unique to China)	High-level company management, rules, structures and/or executive decision making	(Company rules on decision, suggestion, and improvement). 有决策权, 给公司建议, 参与决策管理, 参与提升管理
Actions and procedures (unique to United States)	Concrete actions and activities that can be performed in a workplace context, as opposed to general abstract ideas	Having a meeting, group discussions, welcoming new colleagues, and completing projects
Effort (unique to United States)	A certain level of focus, dedication, effort, attention, etc.	Effortful, hard work, pay attention, and do the best work.

Table 2 shows the most common categories of associations generated by American and Chinese employees, ranked by the frequency mentioned in each culture group. First, the associations of workplace participation generated by American employees were more diverse (in terms of major categories mentioned per employee: *M*_*us*_ = 3.06, *SD*_*us*_ = 1.07) compared with the associations of Chinese employees, which were likely to be focused on a single category [*M*_*cn*_ = 1.36, *SD*_*cn*_ = 0.73; *t*_(240)_ = 16.83, *p* < 0.001, *d* = 2.17]. For the overlapping categories of associations, we used a logistic regression to compare the probability of which American and Chinese employees in our sample would mention each category of associations. Results indicate that American employees were more likely to mention all of the overlapping categories between cultures except for the *Ideology and hierarchy* category (see Supplementary Appendix Table 2 for how results vary based on group membership). In other words, an American employee was more likely to touch base on multiple aspects of workplace participation than a Chinese employee. A Chinese employee was more likely to elaborate on one or two focused dimensions when they thought about workplace participation.

**TABLE 2:** Frequency ranking of categories of associations with workplace participation generated by American and Chinese employees.

Rank	Americans (*n* = 149)	Chinese (*n* = 211)
1	Positivity (64.2%)	Teamwork (35.5%)
2	Teamwork (58.8%)	Positivity (32.7%)
3	Effort (54.1%)	Management rules (27.0%)
4	Actions and procedures (36.5%)	Ideology and hierarchy (16.6%)
5	Extrinsic (27.7%)	Socializing outside of work (13.3%)
6	Ideology and hierarchy (23.0%)	Extrinsic (10.0%)
7	Negative valence (12.2%)	Negative valence (9.0%)

*Numbers in parentheses indicate the percentage of employees who contributed to each category. American employees (M_us_ = 3.06, SD_us_ = 1.07) were more likely to mention multiple categories of participation associations, while Chinese employees [M_cn_ = 1.36, SD_cn_ = 0.73; t_(240)_ = 16.83, p < 0.001, d = 2.17] were more likely to elaborate on one or two focused categories of associations.*

There were both similarities and differences in free associations across the two culture groups. Associations that belong to categories such as *teamwork* and *positivity* were common—both ranked as the top two across cultures. The two culture groups, however, differed in their most frequently generated categories: *Effort, actions and procedures, and extrinsic* came up in the top five for American employees, whereas *management rules, ideology and hierarchy, and socializing outside of work* were among the top five for Chinese employees. Among these categories, *extrinsic* referred to the tangible outcomes of participation such as rewards or costs; both *effort* and *actions and procedures* referred to the process of participation—the former focused on the level of dedication and engagement while the latter focused on concrete activities and actions performed as part of a participatory scheme in a workplace, as opposed to abstract ideas. On the other hand, *management rules* and *ideology and hierarchy* referred to high level abstract associations of workplace participation, while *socializing outside of work* referred to social activities outside a workplace context. The results suggest that American employees’ participation associations were more concrete and action oriented, whereas Chinese employees’ participation associations tended to be more distant (in terms of its relevance to a focal workplace) and more high-level or abstract. In particular, *management rules* was a unique major category that referred to the high-level management philosophy such as the sharing of executive decision making, which was a relatively common association among Chinese employees but rarely mentioned by American employees. *Socializing outside of work* is also a unique major category to Chinese employees, which referred to casual team building activities outside of work, such as group picnic and traveling that was not directly related to work but was done with work teammates. These disparities in participation associations may reflect the difference in culture between the two groups, the prevalence of different workplace participation schemes, and more broadly, how employees understand workplace participation from their own perspectives.

We also found qualitative cross-cultural differences of responses within the same category. For example, when participants expressed negative attitudes toward workplace participation, the Chinese employees frequently expressed a sense of distrust with words and phrases such as ulterior motives (*有内幕*) and cheating (*忽悠*). However, we did not observe the same for the American sample, who used a larger variety of negative words that did not show a clear pattern of negativity. In addition, when participants mentioned concepts related to *ideology and hierarchy*, Chinese employees tended to associate political concepts such as democracy and equality to participation, while American employees used more general terms about workplace hierarchy such as boss and moving up the ladder, which might suggest that workplace participation has been more politicized in China. Interestingly, there was no explicit mention of representative participation forms such as unions, worker representatives, or collective bargaining from employees in either culture group.

#### Further Linguistic Analysis

As a robustness check, we further analyzed the content and valence of employees’ freely generated associations to workplace participation using a word usage counting tool—Linguistic Inquiry and Word Count (known as LIWC, Pennebaker et al., 2001). LIWC is a software program that assesses the occurrence of a word or a category of words in text files and is validated in multiple languages including English and Mandarin Chinese. Each of individuals’ generated associations was formatted as a single plain text file. LIWC reads one word at a time in each target text file and writes more than 80 variables that correspond to the linguistic and psychometric properties of the text file, including summary language variables, linguistic dimensions, grammatical usage, and psychological processes. For the purpose of the current study, we focus on the psychological constructs of the text analysis. The internal and external validity of the psychological constructs (e.g., positive and negative emotions and cognitive strategies) has been assessed in numerous studies and these constructs are generally considered valid across cultures (Tausczik and Pennebaker, 2010).

We used LIWC 2007 Dictionary for the text analysis on both American and Chinese responses. It relies on an internal default dictionary that defines which words should be counted in the target text files and increments a particular domain (e.g., affective processes; also known as sub-dictionaries) when words within such domain are tapped. We are particularly interested in the psychological constructs that include six domains that capture the psychological processes involved in the texts. Unlike the human coding analysis that generated overarching content categories, LIWC text analysis focuses on the common processes that are shared in communication. By tapping into the relative rankings of these common processes in both culture groups, we generate insights into the construal level of employees when they think about participation.

In both groups, the occurrence of perceptual and biological processes was low (see Table 3), which was not surprising as the concept was unlikely to be directly linked to biological and sensory experiences like eating and drinking. The interesting distinction lies in the most heavily focused psychological process in each culture. Affective processes, including the expression of positive and negative emotions, were the most active in American employees’ responses. In other words, American employees associated more emotions and feelings to participation. Specifically, they tended to associate more positive affects (667.68 in Chinese and 1808.44 in the United States) to workplace participation than negative affects (91.32 in Chinese and 342.90 in the United States). This is consistent with the category coding analysis above. Cognitive processes, including abstract level thinking, were the most active in Chinese employees’ responses. This is also consistent with the finding that Chinese employees tended to associate high-level management rules and political ideologies to workplace participation. Social processes were active for employees in both cultures, suggesting a high level of human interaction and collective processes in the concept of workplace participation as people understand it.

**TABLE 3:** Linguistic inquiry and word count (LIWC) text analysis results.

Rank	Americans employees	Chinese employees
1	Affective processes (2155.68)	Cognitive processes (2585.02)
2	Social processes (2150.73)	Social processes (2362.12)
3	Cognitive processed (1989.99)	Relativity (1557.31)
4	Relativity (1394.33)	Affective processes (844.57)
5	Perceptual processes (407.41)	Perceptual processes (144.5)
6	Biological processes (218.24)	Biological processes (102.57)

*Numbers in parentheses indicate LIWC’s calculation of total category frequency based on its text analysis. The Relativity domain refers to the activation of motion, space, and time.*

### Discussion

Using a free association paradigm, Study 1 uncovered qualitative associations with workplace participation in both the United States and China, giving us a better understanding of what workplace participation means from employees’ perspectives. *Positive valence* was frequently mentioned by both culture groups, suggesting a general preference or liking associated with workplace participation. *Teamwork* was the most frequent association in the United States and the second most frequent association in China. This suggests that participation is seen as inherently a group process. In the meantime, there were culturally unique associations—such as *socialization outside of work* and the mention of democracies for Chinese employees and *concrete actions* and *effort* for American employees. Even within a major association category, the two culture groups emphasized different aspects.

These qualitative insights inform the survey development in Study 2. Next, we build on these qualitative insights and prior established scales (e.g., Dundon et al., 2004; Wu and Paluck, 2020) to construct a survey that assesses employees’ preference for workplace participation and their perception of its outcomes with Likert-scale items.

## Study 2

Study 2 tests questions on whether employees from the United States and China would in general prefer a higher level of workplace participation, as well as how both culture groups perceive the normative impact of workplace participation. We focus on three dimensions of workplace outcomes: employee productivity, work satisfaction, and workplace conflict. We hypothesize that while people from both cultures may prefer workplace participation and perceive it as conducive to employee work satisfaction, there may be cultural differences in their perceived benefits of participation to organizational productivity and reduced conflict. The Study 2 of the Chinese survey was pre-registered on AsPredicted^2^.

### Method

#### Participants

United States full-time employees (*n* = 150) were recruited through Amazon Mechanical Turk (Mturk) on August 27th, 2021. 5 participants failed the attention check questions^3^ and were excluded from data analysis. The final sample was comprised of 145 fulltime employees (30.34% women, 69.65% men; *M*_*age*_ = 35.64 years, *SD*_*age*_ = 10.34, range = 22–67 years; 79.31% identified as White or European American, 9.66% Black or African American, 4.83% as Asian or Pacific Islander, and 2.07% Hispanic).

Chinese full-time employees (*n* = 205) were recruited on October 13th, 2021 through Wen Juan Xing, an online crowdsourcing platform in China. No participant failed the attention check questions. The final sample was comprised of 205 fulltime employees (64.88% women, 35.12% men; *M*_*age*_ = 30.51 years, *SD*_*age*_ = 5.79, range = 21–45). Participants were compensated for their participation in the study.

#### Procedure

The survey was administered online through MTurk in the United States and Wen Juan Xing in China. The survey primarily assessed participants’ preferences for different dimensions of workplace participation and their perceptions of participation’s impact on their job outcomes. The survey consisted of four parts: preference for workplace participation (including preference for participatory work decisions, e.g., “I wish to carry out my work in the way I think is the best”; and preference for a flattened hierarchy, e.g., “I’d prefer to work as a partner of my manager as opposed to as a subordinate”; Cronbach’s *a* = 0.74), participation effect on employee productivity (e.g., “Companies that let their employees talk more generally have higher productivity”; Cronbach’s *a* = 0.61), participation effect on employee job satisfaction (e.g., “I feel happier at work when I can express my thoughts on work related issues”; Cronbach’s *a* = 0.72), and participation effect on workplace conflict (e.g., “Companies that let employees talk more would experience more chaos”; Cronbach’s *a* = 0.76). The survey items were measured on a 7-point Liker scale from 1 (strongly disagree) to 7 (strongly agree) for both culture groups. See the Supplementary Appendix for the full survey and descriptive statistics.

Even though productivity, satisfaction, and conflict are well-established constructs in organizational psychology, no established scale is available to directly measure employees’ *perceived effect* of workplace participation on these constructs. In other words, we are interested in the perceived effects, rather than the relative levels of the participation outcomes. We developed the survey items based on past studies on participation and employee voice (Dundon et al., 2004; Wu and Paluck, 2021) as well as the common themes of workplace participation emerged from the Study 1 data. Dundon et al. (2004) conducted a qualitative investigation on managerial interpretations of employee voice and extracted themes such as individual (dis)satisfaction and collective organization. We refined the dimensions that Dundon et al. (2004) extracted based on the free association results in Study 1. For the assessment of participation preference, we focused on participants’ preference for participatory work decisions and hierarchy because these were the dimensions that clearly emerged from the Study 1 data.

In addition, we measured employees’ baseline level of participation in workplace, family, and social life using items adapted from Wu and Paluck (2020), with a 7-point Likert scale (Cronbach’s *a* = 0.73). Sample items include “How often do you follow news about politics, e.g., in the daily newspaper, on television, or on the radio?” and “I speak up and also encourage others go get involved in work meetings.” Demographic data (age, gender, occupation, education, and political alignment) were collected at the end of the survey. All items were translated (with back-translation) into Mandarin by two Chinese–English bilingual speakers and were further refined through informal interviews with an independent convenience sample of Chinese participants with similar demographic information as those in the main study.

### Results

First, we regressed the dependent variables of interest on the culture dummy variable (0 = United States, 1 = China) and a set of demographic variables including gender, education, and occupation background. Consistent with the hypothesis, both Chinese employees (*M*_*cn*_ = 4.69, *SD*_*cn*_ = 0.64) and American employees (*M*_*us*_ = 4.59, *SD*_*us*_ = 0.79) exhibited a general preference to workplace participation, conceptualized as participatory decision making and shared influence between hierarchical superiors and their subordinates at work. The group means were both above the mid-point 4 (neutral) on a 7-point Likert scale, but not much, suggesting an overall slightly positive valence. There was a marginally significant difference in the general participation preference between the two cultural groups [β = 0.14, *t*_(340)_ = 1.72, *p* = 0.09, *d* = 0.19].

For the perceived impact of workplace participation, we did not have explicit hypotheses regarding cross-cultural differences *ex ante* except for perceived productivity and workplace conflict. We used the same analysis strategy and found that contrasting to the hypothesis, Chinese employees reported significantly higher productivity from workplace participation [*M*_*cn*_ = 5.25, *SD*_*cn*_ = 0.80; *M*_*us*_ = 4.98, *SD*_*us*_ = 0.98; β = 0.26, *t*_(340)_ = 2.60, *p* = 0.010, *d* = *0.28*], higher level of job satisfaction from workplace participation [*M*_*cn*_ = 5.39, *SD*_*cn*_ = 0.78; *M*_*us*_ = 4.78, *SD*_*us*_ = 1.22; β = 0.60, *t*_(340)_ = 5.31, *p* < 0.001, *d* = 0.58]. Interestingly, consistent with the hypothesis, we did find that Chinese employees were more likely to associate workplace participation with workplace conflict [*M*_*cn*_ = 4.39, *SD*_*cn*_ = 0.85; *M*_*us*_ = 3.94, *SD*_*us*_ = 1.30; β = 0.42, *t*_(340)_ = 3.43, *p* < 0.001, *d* = 0.37]. See Table 4 for complete regression results.

**TABLE 4:** Study 2 results from linear regression.

	Dependent variable:			
	Preference	Satisfaction	Productivity	Conflict
	(1)	(2)	(3)	(4)
Culture (China = 1)	0.135	0.599***	0.262*	0.418***
	(0.082)	(0.113)	(0.102)	(0.123)
Gender (Female = 1)	0.079	−0.163	−0.095	−0.220
	(0.080)	(0.111)	(0.100)	(0.120)
Education level	−0.045	−0.235***	−0.136*	−0.262***
	(0.047)	(0.066)	(0.059)	(0.071)
Farming	0.039	−0.433	−0.025	−0.248
	(0.319)	(0.442)	(0.400)	(0.479)
Other industries	0.112	0.241	−0.065	0.124
	(0.233)	(0.323)	(0.292)	(0.350)
Production	−0.003	−0.117	−0.041	−0.247
	(0.124)	(0.172)	(0.155)	(0.186)
Sales	−0.018	0.123	−0.005	−0.002
	(0.097)	(0.134)	(0.122)	(0.146)
Service	0.030	0.061	0.088	−0.072
	(0.111)	(0.154)	(0.140)	(0.167)
Education	−0.495	1.886*	1.822*	−1.808
	(0.701)	(0.970)	(0.878)	(1.052)
Baseline participation	0.194***	0.214**	0.181**	0.056
	(0.048)	(0.067)	(0.060)	(0.072)
Constant	3.605***	4.969***	4.779***	2.726***
	(0.360)	(0.498)	(0.451)	(0.540)
Observations	350	350	350	350
*R* ^2^	0.060	0.158	0.074	0.102

*p < 0.05, **p < 0.01, ***p < 0.001. Numbers indicate regression coefficients, with standard errors in parentheses. Results using linear regression. Culture is a dummy variable where China = 1 and United States = 0. Gender is a dummy variable where female = 1 and male = 0. In the occupation variables, “professional and management” serves as a baseline comparison with which “farming,” “other industry,” “production,” “sales,” “service,” and “education” are compared.

Because we estimated four dependent variables from the survey data, we conducted a joint significance test against the null that none of the group difference between China and the United States in these four outcomes were significant. There was a jointly significant difference between the two culture groups in the perceptions and preferences of workplace participation, *F*_(1,348)_ = 8.58, *p* < 0.001.

Furthermore, we tested the correlations between each dependent variable and found significant correlations between participation preference and perceived impact from participation. Consistent with the hypothesis, in both cultures, preference for workplace participation positively predicts the impact of workplace participation. See Table 5 for the descriptive statistics and correlation coefficients in the combined sample (refer to Supplementary Appendix Tables 3, 4 for separate results for China and the United States). With regard to the demographics, employees with lower level of education predicted a larger impact on job satisfaction (β = −0.21, *t*_(340)_ = −3.13, *p* = 0.002, *d* = −0.34) and workplace conflict (β = −0.25, *t*_(340)_ = −3.60, *p* < 0.001, *d* = −0.39) from workplace participation. There was no significant correlation between other demographic variables and participation preference or perceived participation outcomes.

**TABLE 5:** Descriptive statistics and correlations among study variables.

Variable	*M*	*SD*	1	2	3	4	5	6
(1) Preference	4.65	0.70	–					
(2) Productivity	5.14	0.89	0.35***	–				
(3) Satisfaction	5.14	1.03	0.35***	0.65***	–			
(4) Conflict	3.20	1.08	−0.16**	−0.60***	−0.74***	–		
(5) Workplace voice	4.69	1.13	0.34***	0.40***	0.39***	−0.31***	–	
(6) Social voice	5.46	0.81	0.22***	0.13*	0.11*	0.009	0.31***	–

*p < 0.05, **p < 0.01, ***p < 0.001. Workplace voice and social voice refer to individuals’ baseline level of participation at work and outside of work more generally.

### Discussion

Study 2 demonstrates that both culture groups reported a relatively positive level of endorsement of participatory work practices, including participatory decision making and a more flattened hierarchical relationship between superiors and their subordinates. In addition, employees generally associated workplace participation with higher worker productivity and job satisfaction as well as lower workplace conflict.

The current survey design cannot explicitly compare one’s preference of a participatory versus non-participatory workplace. Even though both culture groups expressed endorsement of workplace participation, we cannot rule out the possibility that a non-participatory workplace is similarly endorsed. Next, we conduct a within-subject experiment where we randomly assign employees to mentally interact with a high or low work environment first with repeated measures for its counterpart. Therefore, we can directly compare the perceived desirability of working in each work environment and the downstream outcomes of workplace participation on productivity, job satisfaction, and conflict.

## Study 3

Study 3 tests whether employees from the US and China prefer a participatory work environment to a non-participatory work environment, using an experimental design. We hypothesize that employees in both the US and China generally prefer a participatory work environment to a non-participatory work environment, although cross-cultural differences may exist so that employees from one culture would value a participatory workplace to a greater extent than employees from a different culture. Study 3 was a pre-registered experiment on AsPredicted^4^.

### Method

#### Participants

United States full-time employees (*n* = 205) were recruited through Amazon Mechanical Turk on October 22nd, 2021 (42.44% women, 57.56% men; *M*_*age*_ = 40.12 years, *SD*_*age*_ = 10.91, range = 23–83 years; 74.15% identified as White or European American, 9.76% Black or African American, 9.27% as Asian or Pacific Islander, and 3.90% Hispanic). Chinese full-time employees (*n* = 218) were recruited on October 22nd, 2021 through Wen Juan Xing, an online crowdsourcing platform in China (56.42% women, 43.58% men; *M*_*age*_ = 29.73 years, *SD*_*age*_ = 3.78, range = 26–50 years).

The combined sample consists of 423 full-time employees (49.65% women, 50.35% men; *M*_*age*_ = 37.81 years, *SD*_*age*_ = 11.18, range = 23–83 years). No participant was excluded from the data analysis. Participants were compensated for their participation in the study.

#### Procedure

As pre-registered, we adopted a within-subject repeated measures experimental design. Employees in both the United States and China were presented with two vignettes either depicting a prototypical workplace high in participation (*high-P*) or a prototypical workplace low in participation (*low-P*). We pre-tested the vignettes with an independent sample of 66 online participants to ensure that the workplace environment in the *low-P* vignette was indeed perceived to be significantly lower in workplace participation compared with the workplace depicted in the *high-P* vignette (*p* = 0.027).

Participants were asked to imagine themselves as a mid-level employee in a large corporation that specializes in the manufacturing of household goods and services. They were given vivid depictions of their daily job functions. For example, in the *low-P* condition, the employee would report to work at 9 am and “immediately check-in with your supervisor.” The supervisor would be in charge of most decision making at work. The *low-P* workplace features a highly structured and hierarchical work environment, with little chance for its employees to make decisions or voice opinions at work. In the *high-P* condition, the participants were asked to imagine reporting to work at 9 am “for your all-team group meeting.” Team members would share power and influence at work meetings and in the decision-making processes. The *high-P* workplace features a more democratic work environment with opportunities for its employees to make decisions and voice opinions at work. After engaging with each work environment, participants were asked to rate the extent to which they like this workplace, and their perceived workplace outcomes such as productivity, job satisfaction, and conflict with a 7-point Likert scale from 1 (not at all) to 7 (extremely). Sample items include: “How productive are you at this workplace?”, “How satisfied are you with your daily job functions?”, and “How much workplace conflict do you think you will encounter?”.

The order of the vignette condition presentation was counterbalanced—half of the participants viewed the *low-P* vignette first and the other half viewed the *high-P* vignette first. After the presentation of the first vignette, participants were asked to take a minute to clear their mind before engaging with the second vignette. At the end of the experiment, participants filled out standard demographic questions such as gender, age, education, and occupation.

### Results

As pre-registered, we conducted a repeated measures analysis of variance (ANOVA) where we treated the vignette condition (*low-P* vs. *high-P*) as a within-subject factor and country (China vs. the United States) as a between-subject factor on the main dependent variables: the desirability of each workplace, and perceived outcomes in each workplace, including employee productivity, job satisfaction, and workplace conflict. We were interested to test whether employees in the United States and China would show differential preferences and outcome perceptions comparing a high participation workplace and a low participation work environment.

In the combined sample of American and Chinese employees (*n* = 423), there was a main effect of vignette condition on preference—the high participation workplace was on average rated as more desirable to the low participation workplace [*M*_*high*_ = 4.61, *SD*_*high*_ = 1.27; *M*_*low*_ = 3.96, *SD*_*low*_ = 1.59, *d* = 0.45; *F*_(1,421)_ = 69.04, *p* < 0.001]. However, there was a significant interaction effect between country and condition [*F*_(1,421)_ = 109.22, *p* < 0.001]. For each of the three perceived outcomes, we found significant main effects of condition as well as significant interaction effects between condition and country. In general, employees reported higher productivity [*M*_*high*_ = 5.02, *SD*_*high*_ = 1.08; *M*_*low*_ = 4.91, *SD*_*low*_ = 1.11, *d* = 0.10; *F*_(1,421)_ = 4.34, *p* = 0.04], job satisfaction [*M*_*high*_ = 4.74, *SD*_*high*_ = 1.25; *M*_*low*_ = 4.13, *SD*_*low*_ = 1.57, *d* = 0.43; *F*_(1,421)_ = 61.05, *p* < 0.001], and lower conflict [*M*_*high*_ = 3.21, *SD*_*high*_ = 1.54; *M*_*low*_ = 3.42, *SD*_*low*_ = 1.41, *d* = −0.14; *F*_(1,421)_ = 10.28, *p* = 0.001] in a high participation workplace compared with a low participation workplace.

**FIGURE 1:**
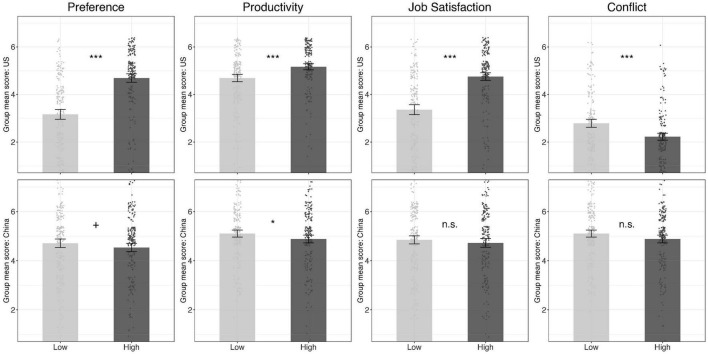
Study 3 results. The figure represents cultural group means for the self-reported *Preference* for each workplace and the perceived outcomes of employee *Productivity*, *Job Satisfaction*, and workplace *Conflict* comparing the *Low-Participation* versus *High-Participation* condition for both the United States (the first row) and the Chinese full-time employees (the second row). Each dot in the background represents an employee’s actual rating on the corresponding construct. Error bars represent 95% confidence intervals. *p < 0.05, **p < 0.01, ***p < 0.001, *n.s.* denotes non-significance with alpha at the conventional 0.05 level.

Culture is clearly a moderator as we found significant interaction effects for each of our primary dependent variables. Therefore, we conducted further analysis for employees in each country to investigate the different patterns (see Figure 1).

#### The United States

For American employees, there was a significant difference in employee preference, where they were significantly more likely to endorse a high participation work environment (*M* = 4.69, *SD* = 1.29) than a low participation work environment [*M* = 3.17, *SD* = 1.49; *F*_(1,204)_ = 144.41, *p* < 0.001, *d* = 1.09]. American employees also perceived different outcomes from a *high-P* workplace versus a *low-P* workplace. Specifically, compared with a *low-P* workplace, employees engaging in the high-P workplace reported significantly higher productivity [*M*_*high*_ = 5.17, *SD*_*high*_ = 0.93; *M*_*low*_ = 4.69, *SD*_*low*_ = 1.12, *d* = 0.47; *F*_(1,204)_ = 33.79, *p* < 0.001], significantly higher job satisfaction [*M*_*high*_ = 4.75, *SD*_*high*_ = 1.16; *M*_*low*_ = 3.36, *SD*_*low*_ = 1.52, *d* = 1.03; *F*_(1,204)_ = 134.52, *p* < 0.001], and significantly lower workplace conflict [*M*_*high*_ = 2.23, *SD*_*high*_ = 1.06; *M*_*low*_ = 2.79, *SD*_*low*_ = 1.23, *d* = −0.49; *F*_(1,204)_ = 36.34, *p* < 0.001].

#### China

For Chinese employees, there was also a difference in employee preference, but the direction was the opposite to that of American employees. The Chinese employees were marginally *less* likely to endorse a high participation work environment (*M*_*high*_ = 4.54, *SD*_*high*_ = 1.25) than a low participation work environment [*M*_*low*_ = 4.71, *SD*_*low*_ = 1.29; *F*_(1,204)_ = 2.86, *p* = 0.09]. There was no statistically significant difference in perceived job satisfaction (*M*_*high*_ = 4.72, *SD*_*high*_ = 1.33; *M*_*low*_ = 4.85, *SD*_*low*_ = 1.24; *p* = 0.26, *d* = −0.10) or workplace conflict (*M*_*high*_ = 4.13, *SD*_*high*_ = 1.34; *M*_*low*_ = 4.02, *SD*_*low*_ = 1.31; *p* = 0.27, *d* = 0.08). However, Chinese employees saw themselves to be significantly *less* productive working in a high participation workplace (*M*_*high*_ = 4.89, *SD*_*high*_ = 1.19) than a low participation workplace [*M*_*low*_ = 5.11, *SD*_*low*_ = 1.07; *F*_(1,204)_ = 6.04, *p* < 0. 01].

Because we tested multiple hypotheses from the Chinese and American survey data, we conducted a joint significance test against the null that none of the group difference between China and the United States in these four outcomes were significant. There was a jointly significant difference *between* the two culture groups in the perceptions and preferences of workplace participation in general, *F*_(1,420_) = 92.04, *p* < 0.001, and there was a jointly significant difference *within* each culture group comparing the high versus low participatory work environment, *F*_(1,420)_ = 14.81, *p* < 0.001.

### Discussion

When a prototypically high participation workplace was pitted against a prototypically low participation workplace, we found clear cultural differences in employees’ preference and perceived job outcomes working in each environment. We found that American employees showed an ambiguously strong preference to the high participation workplace and predicted positive outcomes from working in such a workplace, while the opposite was true for the Chinese employees. Chinese employees were marginally more likely to prefer a low participation workplace and associated it with higher productivity (but not higher job satisfaction or conflict). Culture was clearly a moderator in employees’ preference and perceived impact of workplace participation.

## General Discussion

Across three comparative studies, we explore what the concept of workplace participation entails in the minds of employees from the United States and China as well as their general preference and predicted outcomes of workplace participation. Using a free association paradigm, Study 1 demonstrated cross-cultural similarities and differences in how employees construe workplace participation. In Study 2 with cross-cultural surveys, employees in both the United States and China reported a general preference to workplace participation, and predicted higher productivity, job satisfaction, and lower workplace conflict from workplace participation. Study 3 presented a prototypical high participation work environment and a low participation work environment in a repeated measures experimental design. American employees unambiguously preferred the high participation workplace and reported higher productivity, job satisfaction, and less conflict from it, whereas Chinese employees slightly preferred the low participation workplace and reported higher productivity from it. These findings have several implications.

### Positive Valence and Preference

We found that positive valence seems to be strongly associated to workplace participation for both culture groups in Study 1, indicating a cross-cultural positive association or liking for a participatory work structure. Study 2 further reinforces the general positive valence associated with workplace participation: Chinese and American employees expressed similarly high level of endorsement for workplace participation, conceptualized as participatory decision making and a more flattened group hierarchy at workplace. The perceived impact of workplace participation was positive in both cultures. Furthermore, the self-reported preference for participation was positively correlated with perceived outcomes from participation: those who preferred workplace participation were more likely to think that participation would increase worker productivity and job satisfaction as well as reduce workplace conflict. This is consistent with prior research that self-reported participation level tends to correlate with self-reported job satisfaction and productivity (Weber et al., 2020).

These findings might be explained by theories in procedural justice and self-determination. The general positive valence associated with workplace participation is consistent with the postulation from self-determination theory that employees may have innate psychological needs for autonomy and relatedness and prefer procedures that fulfill these needs. Workplace participation is commonly associated with teamwork and social interactions (from Study 1), which exemplifies relatedness. Participation procedures commonly involve the shared influence between hierarchical superiors and their subordinates, exemplifying increase autonomy within the group. The nature of participation might be closely related to the sense of relatedness and autonomy that employees are seeking in a workplace. Therefore, we observe a general preference and positive perceptions of workplace participation across cultures. In addition, the positive correlation between participation preference and perceived participation outcomes (e.g., job satisfaction and productivity) might be explained by procedural justice: when employees perceive a decision procedure to be fair and just, they are more likely to endorse its outcomes and gain higher motivation as a group (Thibaut and Walker, 1975; Tyler and Blader, 2003). Here, satisfaction may be a more proximal indicator of participation, compared with productivity, which tends to be more distal and depend on other working conditions (Demerouti et al., 2001).

### Different Associations With Workplace Participation

There were noticeable differences in how employees construe workplace participation across cultures. American employees were more likely to mention concrete activities and actions as well as levels of effort and engagement in a workplace context, which we did not observe in Chinese employees’ responses. The frequent mention of day-to-day workplace activities and effort levels from American employees indicates that a participation scheme may be seen as more routinized and familiar to American than to Chinese employees. In contrast, Chinese employees were more likely to describe participation using high-level abstract rules at the company level, suggesting that the concept of participation appeared to be more detached from day-to-day job functions in China. In addition, workplace participation frequently evokes associations to socializing activities outside work, which suggests employees from China, an interdependent culture, may tend to draw a looser boundary between professional and casual settings and infer a broader sense of collectivity from the concept of participation. The associations with casual social activities outside of a workplace as well as high-level management rules did not appear in the American sample.

We also found some culturally unique associations within a given category. For example, when expressing negativity about participation, Chinese employees tended to express distrust while American employees expressed more generic negativity. In addition, when talking about hierarchy, Chinese employees almost always talked about democracy (*民主*) while there was no explicit mention of democracy among the American employees. The findings may reflect the larger societal norms or lack thereof around participation and participatory democracy. Research from scarcity suggests that concepts associated with a valued scarce resource (e.g., time, money, and social influence) would become more accessible when one experience prolonged deprivation of such resource (Mullainathan and Shafir, 2013). Thus, it is possible that the lack of a democratic norm in a Chinese society writ large made the link between participation and democracy more explicit, whereas the default democratic norm for the Americans did not activate the link as much.

Regarding workplace conflict, Chinese employees were more likely to predict a higher level of conflict from participation in Study 2. Intragroup conflict is a well-studied area within organizational psychology (De Wit et al., 2012; Greer et al., 2017). Past literature distinguishes multiple forms of intragroup conflict, such as relationship conflict or disagreements about interpersonal issues within a group, task conflict or disagreements about the content and outcomes of tasks being performed within a group, and process conflict or disagreements about the logistics of task accomplishments (Jehn and Bendersky, 2003; De Wit et al., 2012). Workplace conflict as measured in the current study is closest to relationship conflict. So we discuss the conflict results from the relationship conflict perspective. Correlational analyses in both culture groups indicate negative associations between perceived participation effect on workplace conflict and individual outcomes such as productivity and satisfaction, consistent with prior findings of stable negative relationships between relationship and process conflict and group outcomes (De Wit et al., 2012). However, the higher level of perceived participation effect on conflict from Chinese employees suggest that culture might play a role in the perception of workplace conflict. Apart from cultural context, other group level contextual characteristics such as task type and co-occurrence of conflict types might also moderate the relationship between participation and conflict.

### Norms of High vs. Low Workplace Participation

When asked to interact with two work environments that drastically differed in workplace participation, American employees unambiguously preferred the high participation work environment, while Chinese employees tentatively preferred the low participation work environment. This seems contradictory to Study 2’s finding that both culture groups endorsed workplace participation. However, it is worth noting that Chinese employees’ ratings for both types of workplaces were above the mid-point 4 (neutral), while their ratings for the low participation workplace were marginally higher compared with their ratings for the high participation workplace. This is different from the American employees who rated the low participation workplace as significantly lower than the neutral mid-point. In fact, American employees consistently rated the high participation workplace as more conductive to all measured outcomes, including productivity, job satisfaction, and reduced conflict. Interestingly, Chinese participants reported significantly higher work productivity after interacting with the *low* participation workplace. This points to the advantage of a repeated measures experimental design: even though a high participation workplace is perceived favorably by both culture groups, a low participation workplace is perceived even more so among the Chinese employees but not the American employees.

This may be related to the Study 1 results where the Chinese employees made less concrete mental associations of workplace participation, compared with the American employees who associated participation with specific activities in the workplace. The results suggest that it might be more difficult for the Chinese employees to translate the concept of workplace participation into a tangible and practical work scheme that incorporates into their daily routines, while American employees associated participation with specific actions that may have already been implemented in the workplace. It is possible that the slight preference to a low participation work environment reflects the familiarity to the default work environment that they encounter.

This study joins a burgeoning area of organizational democracy research. The present study addresses a relative scarce line of inquiry within this area, which focuses on employees’ attitudes and perceptions of workplace participation and empowerment (Kahnweiler and Thompson, 2000; Greasley et al., 2005; Jeppesen et al., 2011; Khandakar et al., 2018). The present study represents one of the few comparative studies on organizational participation in two culture that differ in many dimensions. It uses a combination of qualitative, survey, and experimental methods to investigate how employees understand workplace participation and its associated outcomes. Consistent with prior research on voice and influence, there is no unanimous agreement on what participation represents across individuals and cultural contexts, and individuals may desire participation to a different extent based on their group membership, organizational type, and existing level of influence (Jeppesen et al., 2011; Markey et al., 2013; Platow et al., 2015). Different from prior studies that focus on individual level participation behavior or the general effects of participation, the current study focus on the meaning and normative impact of workplace participation from the standpoint of employees. Cross-cultural differences and similarities were identified regarding employees’ mental representations, individual differences, and outcome attributions.

### Limitations and Future Directions

The present research shows that Chinese employees are less likely to associate workplace participation with concrete activities and more likely to associate it with high-level rules. We proposed that these results might be due to the different baselines of participation in the workplace and in society more generally. However, it might also reflect culture-specific ways of thinking (e.g., construal level, dialectical thinking). A related question is given that employees’ mental representations of workplace participation differed (Study 1), to what extent cross-cultural differences found in Study 2 and Study 3 might be biased by employees’ different mental representations? We speculate that the results from Study 2 were more susceptible to employees’ different understanding of “workplace participation.” Even though we piloted our survey scales to ensure content equivalence, it is still possible that employees interpreted the survey questions differently. We are less concerned about similar biases in Study 3 as high vs. low level of workplace participation was operationalized concretely using vignettes. However, the experimental vignettes presented in Study 3 might limit the ecological validity of the study. Organizations in the real world are rarely on either side of the participation spectrum—they usually adopt a mixed set of participation schemes. While it is valuable to learn employees’ general preference and perceptions of a high vs. low work environment in an experimental setting, more naturalistic studies collecting employees’ perceptions of their actual organizations as well their observed behavior will complement the current set of studies. Future studies should also directly assess the baseline level of participation in Western as well as non-Western societies to understand how participation norms at workplace and the society shape individuals’ narratives about workplace participation.

The extant participation research is heavily centered around Western nations and their allies. The current study focuses on China and the United States. Further tests with a diverse sample across nations and across industries are needed for the generalizability and external validity of any single set of studies (Gantman et al., 2018). The current studies relied on a paid subject pool from online survey companies, which might over-represent certain population demographics. Noticeably, our sample tend to over represent employees who have college degree and are professionals. Even though we controlled for employee demographics in the data analyses, the comparability of results from our paid sample and a nationally representative sample is unknown. Modern subject recruitment strategy such as online crowdsourcing and survey firms has made the collection of human subject data more efficient. Using a wide variety of cognitive behavioral tasks, Crump et al. (2013) demonstrates that the response patterns of Mturk participants are comparable to laboratory or field participants, supporting the reliability of Mturk as a tool of behavioral research (Crump et al., 2013). However, the testing environment of an online sample is not under the researchers’ control and some crowdsourcing platforms suffered from slowing rates of population replenishment (Peer et al., 2017). Crowdsourcing platforms are tremendously useful in exploratory studies and cross-cultural studies, but a more comprehensive set of research with a wide variety of populations is needed to robustly document causality and generalizability of our results.

China can be seen as a boundary condition for the test of workplace participation. On one hand, participation might not be as welcomed by Chinese employees because it is in direct contrast to a hierarchical norm in a largely non-democratic society. On the other hand, workplace participation may present a particularly strong appeal to Chinese employees because the baseline level of participation is not as strong compared to other cultural contexts where participation in social and political decision making is more common. It will be interesting for future studies to further explore how baseline levels of participation in a cultural setting can affect individuals’ preference and perceptions of participation, and how individuals’ preference and perceptions translate into participation behavior in their local workplace and the society at large, with a larger sample and combined with more qualitative ethnographic work.

Lastly, we found that teamwork is among the most frequent associations for employees in both China and the United States. Team seems to be a central part of participation. This is not surprising as groups lay the foundation of our cognition and behavior (Lewin, 1947). Local groups that we identify with, such as our work groups, citizen groups, and religious groups, play an important role in the socialization of our attitudes and behavior. Prior research suggests that participatory group interventions that target work group dynamics can have increase employee productivity and change social attitudes toward authority (Wu and Paluck, 2020, 2021). More research on the role of teams in shaping individual behavior is needed. Intervention research on participation and employee behavioral change should also be sensitive to cultural contexts to maximize receptivity and minimize potential backlash.

## Conclusion

The concept of workplace participation can be construed differently by employees from different cultures. The cultural context in the workplace and in the society may play a role in shaping individuals’ preferences and perceptions of workplace participation. Individuals’ perceptions and preference may also inform us with the goals and approaches that we should prioritize when shaping a more participatory work environment and a more democratic society with an increasingly diverse population.

## Data Availability Statement

The raw data supporting the conclusions of this article will be made available by the authors, without undue reservation.

## Ethics Statement

The studies involving human participants were reviewed and approved by UCLA Institutional Review Board. The patients/participants provided their written informed consent to participate in this study.

## Author Contributions

All authors listed have made a substantial, direct, and intellectual contribution to the work, and approved it for publication.

## Conflict of Interest

The authors declare that the research was conducted in the absence of any commercial or financial relationships that could be construed as a potential conflict of interest.

## Publisher’s Note

All claims expressed in this article are solely those of the authors and do not necessarily represent those of their affiliated organizations, or those of the publisher, the editors and the reviewers. Any product that may be evaluated in this article, or claim that may be made by its manufacturer, is not guaranteed or endorsed by the publisher.

## Footnotes

1 We removed a word count outlier (word count = 886) in the United States dataset, who copied and pasted paragraphs from public sources. When this outlier was included in the United States word count, *M* = 23.56, *SD* = 72.39.

2
https://aspredicted.org/blind.php?x=9PQ_P4H

3 There were two attention check questions mixed in the survey. One question asked participants to select “Somewhat Agree” while the other asked participants to select “Strongly Disagree.” We exclude the 5 participants who failed to respond to either question as directed.

4
https://aspredicted.org/blind.php?x=TYC_JNX

## References

[B1] Argyris C. (1993). *Knowledge for Action: A Guide To Overcoming Barriers To Organizational Change.* Hoboken, NJ: Jossey-Bass Inc.

[B2] Bandiera O. Best M. C. Khan A. Q. Prat A. (2021). The allocation of authority in organizations: a field experiment with bureaucrats. *Q. J. Econo.* 136 2195–2242. 10.1093/qje/qjab029

[B3] Battilana J. (2018). Cracking the organizational challenge of pursuing joint social and financial goals: social enterprise as a laboratory to understand hybrid organizing. *M@n@gement* 21, 1278–1305. 10.3917/mana.214.1278 18052372

[B4] Bunderson J. S. Reagans R. E. (2011). Power, status, and learning in organizations. *Organ. Sci.* 22 1182–1194. 10.1287/orsc.1100.0590 19642375

[B5] Clegg H. A. (1960). *A New Approach To Industrial Democracy.* Oxford: Blackwell.

[B6] Crampton S. M. Wagner I. I. I. J. A. (1994). Percept-percept inflation in microorganizational research: an investigation of prevalence and effect. *J. Appl. Psychol.* 79 67–76.

[B7] Crump M. J. McDonnell J. V. Gureckis T. M. (2013). Evaluating amazon’s mechanical turk as a tool for experimental behavioral research. *PLoS One* 8:e57410. 10.1371/journal.pone.0057410 23516406 PMC3596391

[B8] De Wit F. R. Greer L. L. Jehn K. A. (2012). The paradox of intragroup conflict: a meta-analysis. *J. Appl. Psychol.* 97:360. 10.1037/a0024844 21842974

[B9] Deci E. L. Ryan R. M. (2000). The “what” and “why” of goal pursuits: human needs and the self-determination of behavior. *Psychol. Inq.* 11 227–268.

[B10] Deci E. L. Olafsen A. H. Ryan R. M. (2017). Self-determination theory in work organizations: the state of a science. *Annu. Rev. Organ. Psychol. Organ. Behav.* 4 19–43.

[B11] Demerouti E. Bakker A. B. Nachreiner F. Schaufeli W. B. (2001). The job demands-resources model of burnout. *J. Appl. Psychol.* 86:499. 11419809

[B12] Dessein W. (2002). Authority and communication in organizations. *Rev. Econ. Stud.* 69 811–838. 10.1111/1467-937x.00227

[B13] Deutsch S. (2005). A researcher’s guide to worker participation, labor and economic and industrial democracy. *Econ. Ind. Democr.* 26 645–656.

[B14] Donaghey J. Cullinane N. Dundon T. Wilkinson A. (2011). Reconceptualising employee silence: problems and prognosis. *Work Employ. Soc.* 25 51–67. 10.1177/0950017010389239

[B15] Dundon T. Wilkinson A. Marchington M. Ackers P. (2004). The meanings and purpose of employee voice. *Int. J. Hum. Resour. Manag.* 15 1149–1170.

[B16] Foels R. Driskell J. E. Mullen B. Salas E. (2000). The effects of democratic leadership on group member satisfaction: an integration. *Small Group Res.* 31 676–701.

[B17] Gantman A. Gomila R. Martinez J. E. Matias J. N. Paluck E. L. Starck J. (2018). A pragmatist philosophy of psychological science and its implications for replication. *Behav. Brain Sci.* 41:e127. 10.1017/S0140525X18000626 31064540

[B18] Gollan P. J. Xu Y. (2015). Re-engagement with the employee participation debate: beyond the case of contested and captured terrain. *Work Employ. Soc.* 29 N1–N13.

[B19] Greasley K. Bryman A. Dainty A. Price A. Soetanto R. King N. (2005). Employee perceptions of empowerment. *Empl. Relat.* 27 354–368.

[B20] Greer L. L. De Jong B. A. Schouten M. E. Dannals J. E. (2018). Why and when hierarchy impacts team effectiveness: a meta-analytic integration. *J. Appl. Psychol.* 103:591. 10.1037/apl0000291 29369654

[B21] Greer L. L. Van Bunderen L. Yu S. (2017). The dysfunctions of power in teams: a review and emergent conflict perspective. *Res. Organ. Behav.* 37 103–124.

[B22] Hanushek E. A. Link S. Woessmann L. (2013). Does school autonomy make sense everywhere? Panel estimates from PISA. *J. Dev. Econ.* 104 212–232.

[B23] Harley B. Sargent L. Allen B. (2010). Employee responses to ‘high performance worksystempractices: an empirical test of the disciplined worker thesis. *Work Employ. Soc.* 24 740–760.

[B24] Heller F. (1998). Influence at work: a 25-year program of research. *Hum. Relat.* 51 1425–1456. 10.1177/001872679805101202

[B25] Heller F. (2003). Participation and power: a critical assessment. *Appl. Psychol.* 52 144–163.

[B26] Heller F. Pusic E. Strauss G. Wilpert B. (1998). *Organizational Participation: Myth and Reality.* Oxford: Oxford University Press.

[B27] Hespe G. Wall T. (1976). The demand for participation among employees. *Hum. Relat.* 29 411–428. 10.1177/001872677602900503

[B28] Jehn K. A. Bendersky C. (2003). Intragroup conflict in organizations: a contingencyperspective on the conflict-outcome relationship. *Res. Organ. Behav.* 25 187–242.

[B29] Jensen M. C. Meckling W. H. (1976). Theory of the firm: managerial behavior, agency costs and ownership structure. *J. Financ. Econ.* 3 305–360. 10.1016/0304-405x(76)90026-x

[B30] Jeppesen H. J. Jønsson T. Shevlin M. (2011). Employee attitudes to the distribution of organizational influence: who should have the most influence on which issues? *Econ Ind. Democr.* 32 69–86. 10.1177/0143831x10372432

[B31] Kahnweiler W. M. Thompson M. A. (2000). Levels of desired, actual, and perceived control of employee involvement in decision making: an empirical investigation. *J. Bus. Psychol.* 14 407–427.

[B32] Kessler I. Purcell J. (1996). Strategic choice and new forms of employment relations in the public service sector: developing an analytical framework. *Int. J. Hum. Resour. Manag.* 7 206–229. 10.1080/09585199600000125

[B33] Khandakar M. S. A. Huq K. Sultana S. (2018). Perception of employees regarding participation in decision making and problem solving: a study on different branches of banks in Dhaka city. *ABC Res. Alert* 6 77–90.

[B34] Knudsen H. Busck O. Lind J. (2011). Work environment quality: the role of workplace participation and democracy. *Work Employ. Soc.* 25 379–396. 10.1177/0950017011407966

[B35] Krippendorff K. (1980). *Content Analysis: An Introduction To Its Methodology.* Beverly Hills, CA: Sage.

[B36] Landry D. (2008). *Noble Brutes: How Eastern Horses Transformed English Culture.* Baltimore, MD: JHU Press.

[B37] Latham G. P. Yukl G. A. (1976). Effects of assigned and participative goal setting on performance and job satisfaction. *J. Appl. Psychol.* 61:166. 10.1037/0021-9010.61.2.166

[B38] Lewin K. (1947). Frontiers in group dynamics: II. Channels of group life; social planning and action research. *Hum. Relat.* 1 143–153. 10.1177/001872674700100201

[B39] Liverpool P. R. (1990). Employee participation in decision-making: an analysis of the perceptions of members and nonmembers of quality circles. *J. Bus. Psychol.* 4 411–422. 10.1007/bf01013604

[B40] Locke E. A. Latham G. P. (2002). Building a practically useful theory of goal setting and task motivation: a 35-year odyssey. *Am. Psychol.* 57 705–717. 10.1037//0003-066x.57.9.705 12237980

[B41] Lucio M. M. Stuart M. (2005). ‘Partnership’ and new industrial relations in a risk society: an age of shotgun weddings and marriages of convenience? *Work Employ. Soc.* 19 797–817. 10.1177/0950017005058068

[B42] Mansuri G. Rao V. (2013). *Localizing Development. Does Participation Work?.* Washington, DC: World Bank.

[B43] Marchington M. Wilkinson A. (2005). “Direct participation and involvement,” in *Managing Human Resources: Personnel Management in Transition*, ed. Bach S.. (Oxford: Blackwell), 398–423.

[B44] Markey R. Ravenswood K. Webber D. J. Knudsen H. (2013). Influence at work and the desire for more influence. *J. Ind. Relat.* 55 507–526. 10.1177/0022185613489393

[B45] Miller K. I. Monge P. R. (1986). Participation, satisfaction, and productivity: a meta-analytic review. *Acad. Manag. J.* 29 727–753. 10.1016/j.pmedr.2016.11.007 27981022 PMC5153451

[B46] Mullainathan S. Shafir E. (2013). *Scarcity: Why Having Too Little Means So Much.* Basingstoke: Macmillan.

[B47] Nisbett R. E. Masuda T. (2003). Culture and point of view. *Proc. Natl. Acad. Sci.* 100 11163–11170. 12960375 10.1073/pnas.1934527100 PMC196945

[B48] Pateman C. (1970). *Participation and Democratic Theory.* Cambridge: Cambridge University Press.

[B49] Peer E. Brandimarte L. Samat S. Acquisti A. (2017). Beyond the turk: alternative platforms for crowdsourcing behavioral research. *J. Exp. Soc. Psychol.* 70 153–163. 10.1016/j.jesp.2017.01.006

[B50] Pennebaker J. W. Francis M. E. Booth R. J. (2001). Linguistic inquiry and word count:LIWC 2001. Mahwah, NJ: Lawrence Erlbaum Associates, 2001.

[B51] Perrett R. (2007). Worker voice in the context of the re-regulation of employment: employer tactics and statutory union recognition in the UK. *Work Employ. Soc.* 21 617–634. 10.1177/0950017007082873

[B52] Platow M. J. Huo Y. J. Lim L. Tapper H. Tyler T. R. (2015). Social identification predicts desires and expectations for voice. *Soc. Justice Res.* 28 526–549. 10.1007/s11211-015-0254-6

[B53] Richter F. D. Tjosvold D. (1980). Effects of student participation in classroom decisionmaking on attitudes, peer interaction, motivation, and learning. *J. Appl. Psychol.* 65 74–80. 10.1037/0021-9010.65.1.74

[B54] Ritchie J. B. Miles R. E. (1970). An analysis of quantity and quality of participation as mediating variables in the participative decision making process. *Pers. Psychol.* 23 347–359. 10.1016/j.socscimed.2019.112663 31734480

[B55] Rousseau J. J. (1968). *Politics and the Arts: Letter to M. D’Alembert on the Theatre.* Ithaca, NY: Cornell University Press.

[B56] Royle M. T. Fox G. (2011). The relationship between psychological strain, self-regulation, and informal accountability for others. *Int. J. Manag. Market. Res.* 4 1–18.

[B57] Schuler R. S. (1980). Definition and conceptualization of stress in organizations. *Organ. Behav. Hum. Perform.* 25 184–215.

[B58] Stein R. T. Heller T. (1979). An empirical analysis of the correlations between leadership status and participation rates reported in the literature. *J. Pers. Soc. Psychol.* 37 1993–2002. 10.1037/0022-3514.37.11.1993

[B59] Tausczik Y. R. Pennebaker J. W. (2010). The psychological meaning of words: LIWC andcomputerized text analysis methods. *J. Lang. Soc. Psychol.* 29 24–54. 10.1111/j.2044-8341.2012.02065.x 24217863

[B60] Thibaut J. W. Walker L. (1975). *Procedural Justice: A Psychological Analysis.* Mahwah, NJ: L. Erlbaum Associates.

[B61] Truex R. Tavana D. L. (2019). Implicit attitudes toward an authoritarian regime. *J. Polit.* 81 1014–1027.

[B62] Tyler T. R. Blader S. L. (2003). The group engagement model: procedural justice, social identity, and cooperative behavior. *Pers. Soc. Psychol. Rev.* 7 349–361. 10.1207/S15327957PSPR0704_07 14633471

[B63] Tyler T. R. Lind E. A. (1992). A relational model of authority in groups. *Adv. Exp. Soc. Psychol.* 25 115–191.

[B64] Vroom V. H. Yetton P. W. (1973). *Leadership And Decision-Making*, Vol. 110. Pittsburgh: University of Pittsburgh Pre.

[B65] Wagner I. I. I. J. A. Gooding R. Z. (1987). Effects of societal trends on participationresearch. *Admin. Sci. Q.* 241–262. 10.2307/2393128

[B66] Webb S. Webb B. (1902). *Industrial Democracy.* Harlow: Longmans, Green and Company.

[B67] Weber W. G. Unterrainer C. Höge T. (2020). Psychological research on organisationaldemocracy: a meta-analysis of individual, organisational, and societal outcomes. *Appl. Psychol.* 69 1009–1071.

[B68] Wilkinson A. Gollan P. J. Marchington M. Lewin D. (eds) (2010). *The Oxfordhandbook of Participation In Organizations.* Oxford: Oxford University Press.

[B69] Wu S. J. Paluck E. L. (2020). Participatory practices at work change attitudes and behavior toward societal authority and justice. *Nat. Commun.* 11 1–8. 10.1038/s41467-020-16383-6 32457373 PMC7250830

[B70] Wu S. J. Paluck E. L. (2021). *Having a Voice In Your Group: Increasing Productivity Through Group Participation.* Available online at: https://ssrn.com/abstract=3933505 (accessed September 30, 2021).

[B71] Wu S. J. Bai X. Fiske S. T. (2018). Admired rich or resented rich? How two cultures vary in envy. *J. Cross Cult. Psychol.* 49 1114–1143. 10.1177/0022022118774943

