# Supplementary material for: Preferences and Perceptions of Workplace Participation: A Cross-Cultural Study
Source: Front Psychol. 2022 Feb 14;13:806481. doi: 10.3389/fpsyg.2022.806481 (PMC8882961; doi:10.3389/fpsyg.2022.806481)
Supplement: Supplementary file 1 [file Data_Sheet_1.docx]

Appendix

**Table S1.**

*Study 1 Demographic Summary for the Cross-Cultural Sample*

|  | Study1 | | Study2 | | Study3 | |
| --- | --- | --- | --- | --- | --- | --- |
|  | The United States  n = 149 | China  n = 211 | The United States  n = 145 | China  n = 205 | The United States  n = 205 | China  n = 218 |
| Gender | 40.3% female | 55.9% female | 30.3% female | 64.9% female | 42.4% female | 56.4% female |
| Age | 38.6 ± 12.0 | 32.9 ± 7.6 | 35.6 ± 10.3 | 30.5 ± 5.8 | 40.1 ± 10.9 | 29.7 ± 3.8 |
| Race/ethnicity | 7.4% Asian or Pacific Islander  8.7% Black or African American  4.0% Hispanic or Latinx  73.8% White or Caucasian  6.2% Multiracial or Biracial | —— | 4.8% Asian or Pacific Islander  9.7% Black or African American  2.1% Hispanic or Latinx  79.3% White or Caucasian  2.8% Multiracial or Biracial | —— | 9.3% Asian or Pacific Islander  9.8% Black or African American  3.9% Hispanic or Latinx  74.2% White or Caucasian  2.9% Multiracial or Biracial | —— |
| Education  (modal response) | Bachelor’s Degree in College  (n = 78; 52.3%) | Bachelor’s Degree in College (n = 147; 69.7%) | Bachelor’s Degree in College  (n = 90; 62.1%) | Bachelor’s Degree in College (n = 156; 76.1%) | Bachelor’s Degree in College  (n = 84; 41.0%) | Bachelor’s Degree in College (n = 170; 78.0%) |
| Occupation  (modal response) | Professional and Related  (n = 51; 34.2%) | Professional and Related  (n = 100; 47.3%) | Professional and Related  (n = 68; 46.9%) | Professional and Related (n = 95; 46.3%) | Professional and Related  (n = 101; 49.3%) | Professional and Related (n = 110; 50.5%) |
| Conservatism | 3.5 ± 1.8 | 2.9 ± 1.0 | 3.7 ± 2.2 | —— | 3.5 ± 1.8 | —— |

*Note.* “Conservatism” refers to a survey item measuring political orientation, where 1 = *extremely liberal* to *7= extremely conservative.* Reported with mean and standard deviation, otherwise indicated.

**Table S2.**

Descriptive Statistics and Correlations Among Study Variables (United States)

| Variable | *M* | *SD* | 1 | 2 | 3 | 4 | 5 | 6 |
| --- | --- | --- | --- | --- | --- | --- | --- | --- |
| 1. Preference | 4.90 | .79 | __ |  |  |  |  |  |
| 2. Productivity | 4.98 | .98 | .46­^***^ | __ |  |  |  |  |
| 3. Satisfaction | 4.78 | 1.22 | .40^***^ | .69^***^ | __ |  |  |  |
| 4. Conflict | 2.94 | 1.30 | .18^*^ | .62^***^ | .78^***^ | __ |  |  |
| 5. Workplace voice | 4.69 | 1.14 | .44^***^ | .52^***^ | .40^***^ | .32^***^ | __ |  |
| 6. Social voice | 5.47 | .86 | .24^***^ | .08 | -.01 | -.18^*^ | .25^**^ | __ |

*Note.* ^*^*p* < .05, ^**^*p* < .01, ^***^ *p* < .001. Workplace voice and social voice refer to individuals’ baseline level of participation at work and outside of work more generally.

**Table S3.**

Descriptive Statistics and Correlations Among Study Variables (China)

| Variable | *M* | *SD* | 1 | 2 | 3 | 4 | 5 | 6 |
| --- | --- | --- | --- | --- | --- | --- | --- | --- |
| 1. Preference | 4.68 | .64 | __ |  |  |  |  |  |
| 2. Productivity | 5.24 | .80 | .21^**^ | __ |  |  |  |  |
| 3. Satisfaction | 5.39 | .78 | .27^***^ | .60^***^ | __ |  |  |  |
| 4. Conflict | 3.39 | .85 | .12 | .57^***^ | .64^***^ | __ |  |  |
| 5. Workplace voice | 4.70 | 1.12 | .26^***^ | .31^***^ | .44^***^ | .33^***^ | __ |  |
| 6. Social voice | 5.45 | .77 | .20^**^ | .19^**^ | .34^***^ | .19^**^ | .37^***^ | __ |

*Note.* ^*^*p* < .05, ^**^*p* < .01, ^***^ *p* < .001. Workplace voice and social voice refer to individuals’ baseline level of participation at work and outside of work more generally.

Appendix B. Study 1 Coding Procedure

After the free association results were collected, two research assistants fluent in English first independently categorized the qualitative data by identifying the common themes in the participant responses. For example, the responses of “completing a project” and “showing up” may be identified to contain the common theme of procedural thinking since they both suggest a concept of a concrete action. The two RA’s then met to find the overlaps of the themes that they each identified independently to ensure interrater reliability. The overlapping themes were then incorporated into the shared coding scheme that they both used to code the data upon agreement. When selecting themes for the shared coding scheme, we used the following standards: 1) each theme should be prevalent enough in the dataset (i.e., identified by both raters for at least 10% of participants—20 participants—in the entire dataset); 2) each theme should be conceptually distinct from one another. That is, no two themes should have any overlaps in the concepts that they assessed. For example, a theme of “money” and a theme of “extrinsic rewards” identified from the dataset would be considered conceptually overlapping because money could be a form of extrinsic reward.

To differentiate how strongly participants associated each theme with workplace participation, the raters coded the data with three levels of measurement from 0 to 2: A theme in the coding scheme was marked “0” when none of the participant’s responses contained that theme. A theme was marked “1” when fewer than half of the individual participant’s association items contained the theme, and so on. With these guidelines and a shared coding scheme, the research assistants were trained with the scheme and independently coded through the data before meeting to address the discrepancies. The coding scheme was refined through discussion until the interrater reliability was at least .8 for every theme. For the disagreements, an average of the ratings of the two raters was taken when analyzing the frequency of each theme. The same process was repeated with two native mandarin-speaking research assistants when analyzing data from the Chinese sample.
